# Application of Chemical Sensors and Olfactometry Method in Ecological Audits of Degraded Areas

**DOI:** 10.3390/s21186190

**Published:** 2021-09-15

**Authors:** Andrzej Kulig, Mirosław Szyłak-Szydłowski, Marta Wiśniewska

**Affiliations:** Faculty of Building Services, Hydro and Environmental Engineering, Warsaw University of Technology, 20 Nowowiejska Street, 00-653 Warsaw, Poland; Andrzej.Kulig@pw.edu.pl (A.K.); Miroslaw.Szydlowski@pw.edu.pl (M.S.-S.)

**Keywords:** ammonia, degraded areas, ecological audits, gas detector, hydrogen sulphide, odorant concentration, olfactometry, VOCs

## Abstract

Mineral excavation is a common process throughout the world. The open pits remaining after the closure of a mine require well-considered and meticulous reclamation activities aimed at restoring the environmental properties of a given area. The inspections carried out in Poland indicate numerous irregularities in implementing the reclamation process. The research in this study was conducted in six measurement series and includes both chemical and olfactometry determinations by devices: multisensor portable gas detector and field olfactometer. Statistical analysis of the results obtained show high concentrations in ambient air of both chemical compounds (NH_3_, VOCs, H_2_S, CH_3_SH) and odour, excluding the possibility of occurrence in the pit of only waste types contained in the administrative decision on reclamation. In addition to the unpleasant odour, the listed compounds can have dangerous effects on the health and life of living organisms. This paper presents a suitable method of control and detection of irregularities in the conducted processes. The main advantage is the relatively low cost of purchasing sensors and field olfactometers compared to other devices, and the possibility to test the polluted air in situ, without the risk of chemical processes occurring during transport of gas samples to the laboratory.

## 1. Introduction

The exploitation of natural aggregate deposits causes significant disruption of ecosystems. However, mining activities have become inevitable due to population growth and intensive industrial and technological development [1]. Adverse effects of human activities cause transformation of geological, hydrological, soil and relief conditions and reduced biodiversity. The Sustainable Development Goals (SDGs) [2], developed a few years ago by the United Nations (UN), include land management and restoration [3]. 

Reclamation work on disturbed areas is essential in preserving natural resources and maintaining sustainable use [4,5,6]. The direction of reclamation depends on many parameters such as the slope of the pit walls, permeability of the material for water and plant roots, the scale of erosion, soil type and its grain size, pH, existing fauna and flora species and historical and landscape values [7,8]. The most common direction for reclamation of pits is forestry and agriculture (cultivation, breeding) [7]. However, the reclamation of such areas may also take such directions as recreational (leisure and tourist, sports, cultural), water (recreational, economic, fishing, nature) and didactic (e.g., educational paths) [9,10,11].

The reclamation process consists in restoring a degraded site’s natural and amenity values. It is essential that reclamation activities do not have a negative impact on the environment. The reclamation involves levelling a larger area of land, sometimes in combination with a change of soil type. To fill in the excavation pit, i.e., to carry out the process of macrolevelling, materials compatible with recommendations for the reclamation of the given area are used, e.g., in the form of waste that does not adversely affect the environment of the given excavation pit and its surroundings, such as waste from construction, renovation and disassembly of buildings and road infrastructure (including soil from contaminated areas), byproducts of combustion processes or the final products of stabilization of biodegradable waste separated mechanically from the stream of mixed waste [12,13,14].

In Poland, about 40 types of minerals are mined using open-pit methods, which include mainly sands, gravels, clays, building stone and road construction, limestone and brown coal [15]. In accordance with the law in force in Poland, owners of post-mining excavations are obliged to carry out the reclamation (recultivation/rehabilitation) of the excavated pit, i.e., to restore the natural and functional values of the environment [7,16,17].

In the European Union countries, the land owner is obliged to correctly reclaim the post-mining land following the administrative decision issued. The inspections carried out in Poland by the Supreme Chamber of Control indicate numerous irregularities in implementing the reclamation process. An overall process is backfilling of waste of various origins in excavations, including municipal and hazardous waste [18].

The compounds characteristic for organic matter decomposition processes occurring at municipal waste management plants (mechanical–biological waste treatment plants (MBTP), municipal waste biogas plants (MWBP) and landfills) include mainly alkanes, alkenes, ketones, terpenes and a broad spectrum of volatile organic compounds (VOCs) [19,20,21,22,23]. Some of these substances are formed by decomposition processes of sulphur- and nitrogen-containing compounds. Many are characterized by unpleasant odour [24]. Chemical compounds that produce an olfactory sensation are called odorants [25].

Globally, nearly 70% of the waste generated is disposed in landfills [26,27]. Since the introduction of the Landfill Directive by the European Union in 1999, there has been a yearly reduction of biodegradable waste going to landfills in the member states, which translates into a decrease in odorant emissions from landfills [28,29].

Municipal waste landfills, in contrast to hazardous waste landfills, are well recognized in the literature in terms of the processes occurring in the deposited waste and process gas emissions. The latter are mainly related to the decomposition of organic matter in the deposited waste [30]. Landfill gas consists mainly of methane (CH_4_, 50–60%), carbon dioxide (CO_2_, 40–50%), but also, as demonstrated by Young and Parker [31], hundreds of various chemical compounds present in trace amounts, the so-called trace gases (1%). Landfill trace gases can be divided into organic and inorganic sulphur compounds, alcohols, ketones, aldehydes, esters and ethers, acetates and acids, furans, volatile fatty acids (VFAs), aromatic hydrocarbons (aromatic compounds containing one or more benzene rings), halogenated compounds (compounds containing F, Cl or Br), terpenes (a subgroup of hydrocarbons with five carbon atoms) and nitrogen-containing compounds including ammonia [32,33,34,35]. Many of these compounds, in addition to their unpleasant odour, are also harmful to the health of living organisms, and to the environment.

There are many methods for the quantitative analysis of emitted process gases, including the quantitative analysis of odour. These methods can be generally divided into laboratory methods, which require sampling a gas using a unique bag and its determination under laboratory conditions, and field methods. Both the sampling and its resolution are performed at the source (in the field) [36,37].

Field methods allow collection of a larger number of gas samples and the results are obtained immediately, and therefore not subject to the risk of chemical reactions occurring in the sample during transport time to the laboratory [38,39]. For the quantification of specific compounds, portable gas detectors equipped with sensors are very often used, which are dictated by relatively low investment costs compared to other types of devices and uncomplicated operation [40]. Chemical sensors are used for environmental studies and they are also applied to visualize fruit crop quality indicators [41]. Comparing high-capacity gas detectors with gas chromatographs, the latter are much more extensive devices, requiring longer response times [42,43]. The sensor types available on the market differ mainly in their operating principle, sensitivity, detection range, response time, gas flow rate and sensitivity to temperature and relative air humidity [44].

In the literature, one can find, among others, descriptions of various types of chemical sensors, such as photoionization detectors (PID), flame photometric detectors (FPD) and far UV absorbance detectors (FUV), used mainly in gas chromatography, and electrochemical detectors (EC) (potentiometric—measurement of voltage, amperometric—measurement of current and conductometric—measurement of conductivity) [45,46].

Olfactometers are used to quantify odour. Dynamic laboratory olfactometry is a standardized method (European Standard EN 13725:2003 Air Quality—Determination of Odour Concentration by Dynamic Olfactometry [47]) in contrast to field olfactometry. Odour measurements carried out with olfactometers of St Croix Sensory Inc., Olfasense or Scentroid, meet the requirements of the European Standard [47] in terms of odour measurement approach based on the YES (odour is perceptible)/NO (odour is not detectable) method [48,49].

The application of chemical sensors and sensory methods enable monitoring technological processes in waste management plants, demonstrated in [20,50]. This research aims to verify whether chemical air quality tests can, together with olfactometric analyses, have practical application in ecological audits of the areas. Moreover, an attempt was made to check the applicability of the sensor method as a tool for detecting irregularities in the recultivation carried out, including macrolevelling of post-mining excavations. Furthermore, consequently, to check whether olfactometric testing (with particular emphasis on in situ testing) in combination with sensory, field chemical analysis provide an appropriate, adequate technique for monitoring of some results, including accurate land reclamation results.

The novel contribution of the present research is the combination of field olfactometry with field studies of chemical compounds using electronic sensors to assess the condition of a site after reclamation. These tests, supplementing the classical determinations of soil and groundwater pollutants concentrations in degraded areas, allow for complex analysis of the state of the environment and undesired emission of compounds, treating this emission holistically and considering odour–odorant relations. These methods allow for a larger number of samples than laboratory methods. In addition, results are obtained immediately, and sampling points can be adapted to current results. The last of these aspects is particularly important in the case of a heterogeneous object such as the landfill studied, where the locations of odorogenic compound emissions were unpredictable and their location could not be determined a priori. Only the results obtained in real-time allowed for locating some of the significant odour sources. This complementary approach of field studies is a novelty in assessing the effects of remediation of degraded sites.

## 2. Materials and Methods

The tests were carried out in the eastern part of the Masovian Voivodeship in the area of the excavation (former gravel pit), which was filled with waste and covered with a layer of soil. Materials that could be used legally to fill in the excavation included, among others, waste from mining of minerals other than metal ores, waste sands and clays, solid sediments from beet cleaning and washing, concrete waste and brick rubble from demolition and repair works. 

Chemical tests, including the determination of odorant concentrations of volatile organic compounds (VOCs), ammonia (NH_3_), hydrogen sulphide (H_2_S) and methanethiol (CH_3_SH), which are the characteristic compounds, for the processes of decomposition of organic matter, were performed with a MultiRae Pro gas detector (RAE Systems, Inc., San Jose, CA, USA). In addition, the percentage of methane (CH_4_) was determined using a MultiRae Lite gas detector (RAE Systems, Inc., San Jose, CA, USA). At each of the measurement points, measurements of the analyzed compounds were repeated five times. The characteristics of the substances determined are presented in Table 1.

Olfactometric studies were performed using portable field olfactometers of the Nasal Ranger^®^ and Scentroid SM-100 types. They allow the creation of calibrated dilution series by mixing odorant-contaminated air with filtered, odorant-free air. Field odour nuisance testing using field olfactometers is a method of quantifying odour concentrations in dilution-to-threshold ratios (D/T). The D/T value is the number of dilutions required to achieve a dilution in the ambient air that the odour is undetectable. Owing to the application of these olfactometers, it is possible to calculate the D/T value and, on its basis, the odour concentration value, determined as c_od_ in odour units (ou—odour unit, analogically to the European standard [46]) per volume unit [ou/m^3^] [36]. Temperature (T, °C) and relative humidity (RH, %) were measured using a Rotronic HydroPalm psychrometer with HygroClip2 HC2-S3 sensor. Measurements were conducted at the height of 1.5 m.

One-way ANOVA was used for statistical tests and the Games–Howell post hoc test was used to determine which series were statistically significantly different. This test is used to compare all possible combinations of group differences when the assumption of homogeneity of variances is violated. The Games–Howell method is an improved version of the Tukey–Kramer method and is applicable in cases where the equivalence of variance assumption is violated. It is a *t*-test using Welch’s degree of freedom. This method uses a strategy for controlling the type I error for the entire comparison and is known to maintain the preset significance level even when the sample size differs [51]. Classical regression generalized linear model with Gaussian distribution was performed. 

The research in this study was conducted in six measurement series between January and August 2020, including one pilot series. The schedule of the measurement series carried out is shown in Table 2. 

**Figure 1:**
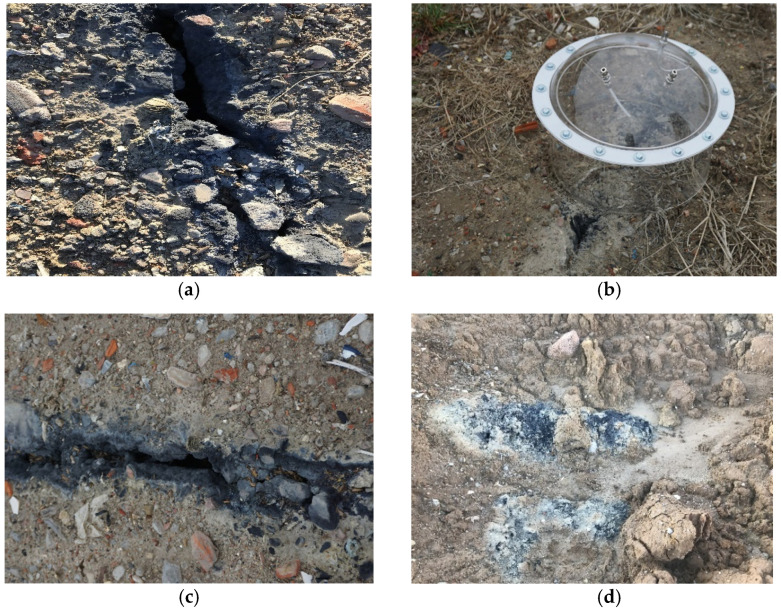
Distinctive sites in the study area indicative of significant degradation (**a**,**b**—photographs taken in 2019; **c**,**d**—photographs taken in 2020).

The first series of measurements (with 17 receptor points) was of a pilot character and included reconnaissance of the studied area, the preliminary determination of odour emission sources and identification of discolourations and cracks in the top layer of the reclamation cover. Figure 1 contains photographs of individual, characteristic sites located in the study area.

During the pilot studies, it was found that the temperature of gases emerging from cracks in the ground was as much as 20 °C greater than the temperature of the ambient atmospheric air. It depended on the location and size of the fissure in the surface of the reclaimed excavation. A static nonventilated hood was used for sampling in this series to take the escaping gases directly from the fissures, minimizing the influence of external conditions on the content of the analysed compounds. Another five series were performed at 75 points as spatial distributions of concentrations in ambient air (without the use of a static hood).

## 3. Results and Discussion

**Figure 2:**
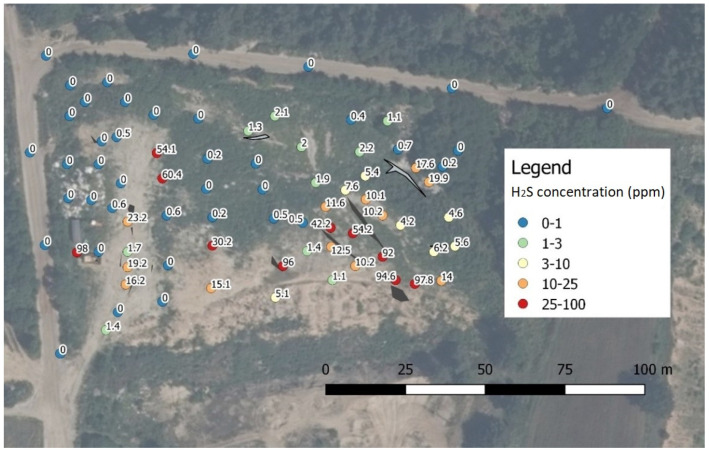
Spatial distribution of hydrogen sulphide concentration in series 2.

The statistical analysis, which is presented later in this paper, shows that the most representative spatial distribution of H_2_S was observed in series 2, due to the strongest correlation with odour concentration This distribution is shown in Figure 2. Table 3, Table 4, Table 5 and Table 6 show the results of odorant and odour concentrations. The measurement results of methane are not presented, since every determination was below the quantification threshold, which may indicate that the source of gas emissions is not typical municipal waste; methane in biogas emitted from municipal landfills usually represents between 50% and 60% of the mixture [29,30].

**Figure 3:**
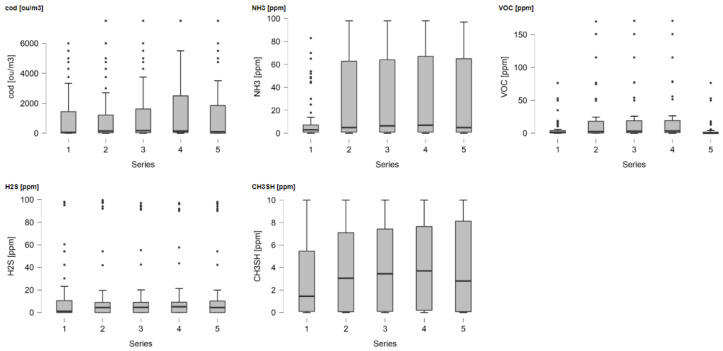
Boxplots of compound concentrations in the five series (time points).

The results obtained in this study were visualized using Esri ArcMap software and analyzed statistically by StatSoft Statistica 13.1 and R 4.1.0 software. Descriptive statistics of the results achieved in all measurement series are presented in Table 3, while Figure 3 contains boxplots of compound concentrations.

In the first series of tests, we found much lower ammonia concentrations than in the other series; a similar relationship was observed for mercaptans and VOCs. In the latter case, maxima were marked in series 2, 3 and 4, while in series 5 the value of VOCs concentration decreased. This may indicate a reduced intensification of odorogenic compounds during the decomposition of the deposited waste. This emission was strongest in series 2, 3 and 4 of the study. Therefore, these results prove that there is a valid reason to believe that the pit was not filled correctly, because, especially in series 2–4, there was a strong emission of odorogenic compounds in health-threatening concentrations.

The high concentrations of the compounds tested may indicate differences between the designed and actual macronutrients. Considering the test results obtained, the types of waste deposited in the pit do not meet the criteria for waste that can be subjected to the landfilling process [28]. The results of odorant concentrations may indicate the occurrence of thermal processes as well as chemical and biological reactions. The release of gases from volatile compounds contained in the deposited waste occurs due to increased temperature [52,53]. The question of the types of waste deposited in the pit is very difficult to resolve. The visible dark colour and cracks on the surface of the investigated area (Figure 1) may be a result of the precipitation reaction of some heavy metals such as lead, mercury, copper, iron, nickel and cobalt in reaction with hydrogen sulphide, which was detected in significant amounts during the conducted studies [54,55,56,57]. The presence of sulphides of these metals may indicate the medical origin of the waste. Toxic compounds present in these wastes pose a significant threat to groundwater and neighbouring drinking water intakes due to the possibility of their infiltration and migration [58].

High concentrations of ammonia and sulphur compounds recorded during the study are also characteristic, e.g., decomposition of organic matter contained in municipal waste disposed in landfills. The decomposition of the organic fraction by microorganisms is cited as the main cause of odour emissions from landfills [59]. For this type of waste, parameters such as C/N ratio, temperature, moisture content and pH can influence the ammonia concentration [20,60,61]. When analyzing the concentrations of volatile sulphur compounds, their level may be determined mainly by the temperature and oxygen concentration [60,62]. In addition, as stated by Bruno et al. [63] and Toledo et al. [64], odour emissions associated with waste treatment odours generated also depend on the type of raw material, the stage of decomposition, and the microclimatic conditions at the treatment site [65].

The literature review indicates that it is difficult to make an unambiguous division when examining proper and improper reclamation filling of an excavation. One of the most important indicators of properly performed rehabilitation of the pits in the research conducted is the nonpollution of the surrounding environment [66]. However, it is generally accepted that properly carried out reclamation should lead to the maintenance of air quality parameters at the background level, i.e., the same as in the surrounding reclaimed area. This means that the area of the former excavation, following reclamation, should not be a source of pollutant emissions to the atmosphere. In the analyzed case, the source of the emission of substances is listed in Table 1. The compounds investigated in the present work are included in such pollutants. Their limit values vary among countries [67]. In Austria, for example, permissible odour concentration levels are regulated through guidelines without legal significance. A concentration of 1 ou/m^3^ is defined as weak odour intensity and a relationship of 1 ou/m^3^ < c_od_ < 8 ou/m^3^ is defined as strong odour intensity [68]. In the case of hydrogen sulphide, there is a low olfactory detection threshold (OT), for example, 0.0081 ppm according to [69]. As it increases, it causes a strong and unpleasant odour. Irritation and nausea are expected at concentrations above 10 ppm, and respiratory and eye damage is expected at concentrations above 50 ppm. In the 300 to 500 ppm range, exposure to H_2_S can be life threatening, and concentrations above 700 ppm are fatal [70,71]. For ammonia, the OT according to [72] is 5.2 ppm. According to the National Institute for Occupational Safety and Health (NIOSH), the maximum permissible time-weighted average (TWA) exposure to anhydrous ammonia for an 8-h workday in a 40-h week is 25 ppm. The short-term exposure limit (STEL), or the concentration at which exposure lasting more than 15 min is potentially hazardous, is 35 ppm. The concentration at which the gas is immediately harmful to life or health (IDLH) is 500 ppm [73,74]. Methyl mercaptan, like hydrogen sulphide, is a volatile sulphur compound (VSC). Compared to H_2_S, it has a much lower OT, 0.00393 ppm, according to [69], but it does not show such harmful effects on the health of living organisms. The situation is much more difficult to interpret in the case of VOCs, which cover a wide range of compounds having carbon in their structure. In the case of studies carried out in biogas plants processing municipal waste, during which besides olfactometry and detector also gas chromatography was used [20], the dominating VOCs were the compounds styrene (OT = 0.047 ppm [75]), phenol (OT = 0.048 ppm [76]) and toluene (OT = 0.33 ppm [77]), which, apart from unpleasant odour, are also characterized by a dangerous effect on human health and life. Analyzing the results presented and the characteristics of the compounds, it can be concluded that the recultivation activities carried out on the site do not comply with the decision issued by the state authority or with the definition of recultivation. The recorded concentrations of odour and chemical compounds cause direct danger to vegetation but are mainly a significant odour nuisance and an indirect threat to the health of living organisms, which is particularly important in the case of nearby settlements and animal habitats. The boxplots in Figure 3 indicate potentially significant differences in ammonium nitrogen concentration between the first time points and subsequent time points in the series. Therefore, one-way ANOVA test was used to determine if these differences were statistically significant (Table 4).

In the case of c_od_ and H_2_S concentration, F-statistic is less than 1, which may indicate that within-group variance is greater than the between-group variance. In this case, it could be a lack of the effect (furthermore, *p*-value is significant, almost 1.0). The desired significance level of less than 0.05 is usually obtained only when the F statistic is at least equal to or greater than two. This case occurs with NH_3_ and VOCs, and the *p*-values are <0.001 and 0.004, respectively. Therefore, one-way ANOVA test was to determine whether there are any statistically significant differences between the means of groups. There is a statistically significant difference between the means of ammonium nitrogen concentration results, so post hoc tests were made (Table 5).

Based on the results of the Games–Howell post hoc test, statistically significant differences were found between the ammonia concentration results determined in the first series and the concentration results in the other series. These results between series 2–5 are not significantly different. Pearson’s correlation matrix among the different analytes for all times series is presented in Table 6.

Upper and lower confidence intervals are constructed so that the designated proportion (confidence level) of these intervals will include the true population value; 95% CI Upper is defined by a limit above the estimated parameter value (0.95), while 95% CI Lower defined by a limit above the estimated parameter value. All correlation results between the tested values are statistically significant (*p* < 0.001). The strongest correlation, with a correlation coefficient value of 0.864, was noted between hydrogen sulphide and odour concentrations. A strong correlation was also noted between hydrogen sulphide concentration and VOCs concentration.

The sensor method used in this study can be compared with the aspiration method (analytical, colorimetric method), which involves extracting specific contaminants when air is passed through a selective filter. The sampling time is related to the concentration averaging time, while the analysis time depends mainly on the type of contaminant and can be up to several hours. Measurement results are averaged over the sampling time, making it impossible to obtain concentration values for very short time intervals, such as a few seconds. The analysis is limited to single chemical compounds whose concentrations in the test gas do not necessarily translate into actual odorous air quality. The results of odorant concentrations may be affected by errors resulting from the operation of particular elements of aspirators (e.g., flow meter), inaccurate pouring of absorbing solutions, preparation of samples for comparison with standards and variable perception of the eye of the person performing the analysis. This method is more appropriate for low gas concentrations (<2 ppm) due to the need to prepare a suitable scale of standards [78]. Based on the experience gained by the authors’ team during aspiration and sensor tests on another site, the results of which have not yet been published, significant differences in the magnitude of the results obtained were found. At high concentrations, up to twice the concentration of the aspiration method was obtained with the sensor method, while at low concentrations the differences between measurements were only about 5%.

## 4. Conclusions

Mineral excavation is common process throughout the world. The open pits remaining after the closure of a mine require well-considered and meticulous reclamation activities aimed at restoring the environmental properties of a given area. In the case of recultivation based on macronivelation, i.e., filling the excavation with specific types of waste, undesired actions harmful to the environment often occur. Lack of adequate supervision during reclamation may result, among others, in uncontrolled emission of odorous and toxic substances.

The research carried out in this study using sensors and olfactometric methods indicates irregularities connected with the way the area was recultivated. High concentrations in ambient air of both chemical compounds (NH_3_, VOCs, H_2_S and CH_3_SH) and odour exclude the possibility of occurrence in the pit of only the types of waste contained in the administrative decision on reclamation. In addition to the unpleasant odour, the compounds listed, especially NH_3_ and H_2_S, but also many types of VOCs, can have dangerous effects on the health and life of living organisms.

The dark colour of the soil and the crevices from which they emit odorous compounds, which may indicate the presence of heavy metal sulphides, are cause for concern. However, the main problem is the lack of knowledge concerning the origin of the waste deposited in the pit and its possible adverse ecological effects.

The statistical analysis of the results obtained indicates a strong correlation between the odorants and odour concentration. The highest correlation was observed between H_2_S and c_od_ concentrations.

Although the methods presented in this paper cannot give an unambiguous answer as to the types of waste used in the process of reclamation of excavations or information on all compounds emitted from them, they are an appropriate method for detecting and monitoring irregularities in the processes conducted, resulting from the observation that a properly rehabilitated area is not a source of high odorant and odour concentrations. In summary, the study confirmed that olfactometric field tests combined with chemical sensor analysis, is an appropriate and adequate technique for monitoring some results, including accuracy of land recultivation results. The main advantage is the relatively low cost of purchasing sensors and field olfactometers compared to devices such as gas chromatographs, but also the possibility to test the polluted air in situ, without the risk of chemical processes occurring during the transport of gas samples to the laboratory.

## Figures and Tables

**Table 1:** Portable gas detector chemical sensors characteristic [20,51].

Compound	Sensor Type	Range	Resolution	Ionization Energy (IE)	Response Time (RT)	Calibration Gas
VOCs	photoionization detector (PID)	0–1000 ppm	0.01 ppm	10.6 eV	15 s	C_4_H_8_ (10.02 ppm; 100 ppm)
NH_3_	electrochemical (EC)	0–100 ppm	1 ppm	Not applicable	60 s	NH_3_ (46.44 ppm)
H_2_S		0–100 ppm	0.1 ppm	Not applicable	35 s	H_2_S (25.8 ppm)
CH_3_SH		0–10 ppm	0.1 ppm	Not applicable	<35 s	CH_3_SH (5 ppm)
CH_4_	protected catalytic bead	0–100%	0.1%	Not applicable	30 s	50% CH_4_

**Table 2:** Schedule of the measurement series.

No	Date	Temperature (°C)	Humidity (%)	Wind Speed (m/s)
		Mean		
1	20 November 2019	2.1	78.4	2.9
2	15 January 2020	0.2	82.3	4.9
3	11 March 2020	6.2	77.5	3.6
4	3 June 2020	14.7	62.8	2.7
5	17 June 2020	20.1	75.2	2.1
6	24 June 2020	17.4	90.1	5.6

**Table 3:** Descriptive statistics of the results achieved in all measurement series.

	Concentration				
	c_od_ [ou/m^3^]	NH_3_ [ppm]	VOC [ppm]	H_2_S [ppm]	CH_3_SH [ppm]
Sample size	380	380	380	380	380
Maximum	7500	98	171	99.6	10.0
Mean	1237	23.8	11.5	13.7	3.89
Median	111	5.00	1.60	2.45	2.85
Minimum	0	0	0.00	0.00	0.00
Standard deviation	2025	32.9	26.2	27.2	3.77

**Table 4:** One-way ANOVA results of concentration values grouped by the number of the series.

Concentration	F	*p*
c_od_ [ou/m^3^]	0.3167	0.867
NH_3_ [ppm]	7.8263	<0.001
H_2_S [ppm]	0.0578	0.994
CH_3_SH [ppm]	1.0628	0.376
VOC [ppm]	3.9615	0.004

Abbreviations: F—Fisher’s statistic coefficient; *p*—*p*-value (test probability).

**Table 5:** Games–Howell post hoc test for ammonium nitrogen results.

Coefficients		Series				
		1	2	3	4	5
1	Mean difference	—	−15.9 **	−16.895 **	−16.9342 **	−15.816 **
	*p*-value	—	0.006	0.003	0.003	0.006
2	Mean difference		—	−0.974	−1.0132	0.105
	*p*-value		—	1	1	1
3	Mean difference			—	−0.0395	1.079
	*p*-value			—	1	1
4	Mean difference				—	1.118
	*p*-value				—	1
5	Mean difference					—
	*p*-value					—

Note: ** *p* < 0.01.

**Table 6:** Pearson’s correlation matrix among the different analytes for all times series.

		Concentration				
Concentration	Coefficients	c_od_ [ou/m^3^]	NH_3_ [ppm]	VOC [ppm]	H_2_S [ppm]	CH_3_SH [ppm]
c_od_ [ou/m^3^]	Pearson’s r	—				
	95% CI Upper	—				
	95% CI Lower	—				
NH_3_ [ppm]	Pearson’s r	0.552 ***	—			
	95% CI Upper	0.619	—			
	95% CI Lower	0.478	—			
VOC [ppm]	Pearson’s r	0.656 ***	0.582 ***	—		
	95% CI Upper	0.71	0.645	—		
	95% CI Lower	0.594	0.512	—		
H_2_S [ppm]	Pearson’s r	0.864 ***	0.567 ***	0.786 ***	—	
	95% CI Upper	0.888	0.632	0.822	—	
	95% CI Lower	0.836	0.495	0.744	—	
CH_3_SH [ppm]	Pearson’s r	0.658 ***	0.632 ***	0.421 ***	0.472 ***	—
	95% CI Upper	0.712	0.689	0.5	0.546	—
	95% CI Lower	0.597	0.568	0.334	0.39	—

Note: *** *p* < 0.001. Abbreviations: 95% CI Upper—upper confidence interval; 95% CI Lower—lower confidence interval.

## Data Availability

Data available on request.
